# Circulating microRNA203 and its target genes' role in psoriasis pathogenesis

**DOI:** 10.3389/fmed.2022.988962

**Published:** 2022-10-20

**Authors:** Sally Abdallah Mostafa, Mai H. S. Mohammad, Walaa A. Negm, Gaber El Saber Batiha, Saqer S. Alotaibi, Sarah M. Albogami, Michel De Waard, Noha Z. Tawfik, Hoda Y. Abdallah

**Affiliations:** ^1^Department of Medical Biochemistry and Molecular Biology, Faculty of Medicine, Mansoura University, Mansoura, Egypt; ^2^Department of Clinical Pathology, Faculty of Medicine, Suez Canal University, Ismailia, Egypt; ^3^Pharmacognosy Department, Faculty of Pharmacy, Tanta University, Tanta, Egypt; ^4^Department of Pharmacology and Therapeutics, Faculty of Veterinary Medicine, Damanhour University, Damanhour, Egypt; ^5^Department of Biotechnology, College of Science, Taif University, Taif, Saudi Arabia; ^6^Smartox Biotechnology, Saint-Egrève, France; ^7^L'institut du thorax, INSERM, CNRS, Nantes University, Nantes, France; ^8^LabEx “Ion Channels, Science & Therapeutics”, Université de Nice Sophia-Antipolis, Nice, France; ^9^Dermatology, Venereology, and Andrology Department, Faculty of Medicine, Suez Canal University, Ismailia, Egypt; ^10^Medical Genetics Unit, Department of Histology and Cell Biology, Faculty of Medicine, Suez Canal University, Ismailia, Egypt; ^11^Center of Excellence in Molecular and Cellular Medicine, Faculty of Medicine, Suez Canal University, Ismailia, Egypt

**Keywords:** MiR-203, miRNAs, psoriasis, SOCS3, SOCS6, TP63, TNF-α, IL8

## Abstract

Numerous microRNAs (miRNAs) have been found to have an aberrant expression in the peripheral blood or psoriasis patients' lesions. Psoriasis was shown to have the abnormal expression of microRNA-203 (miR-203). It is a skin-specific signal that governs cellular proliferation in a protein kinase C-dependent manner and is mostly generated by keratinocytes. This work evaluated the expression levels of the circulating miR-203 target genes SOCS3, SOCS6, TP63, TNF-, IL8, and IL24 in psoriasis patients. Using a relative quantitation PCR technique, we determined the expression levels of miR-203 and its target genes (SOCS3, SOCS6, TP63, TNF-, IL8, and IL24) in the plasma of 120 psoriatic patients and matched healthy controls. The disease characteristics of the patients were then correlated with the expression results. We also conducted numerous enrichment analyses for the diseases, functions, and pathways connected to the under-researched biomarkers. Compared to healthy controls, psoriatic patients had significantly increased levels of miR-203 expression; 7.1 (4.4–9.9). In contrast, psoriatic patients had significantly lower expression of all the examined genes compared to healthy controls. Regarding all the study biomarkers, the receiver operating characteristic (ROC) curve analysis demonstrated significant sensitivity and specificity for differentiating between psoriatic patients and healthy controls. According to the results of the disease matching score generated by miR-203 and its target genes, psoriasis was ranked first with a score of 4.45. The third-place finisher with a value of 3.98, it also demonstrated that miR-203 and its target genes are connected to various skin disorders. Our results show that miR-203 contributes to psoriasis pathogenesis not only locally in skin lesions but also in circulation, indicating that it may contribute to the systemic symptoms of the illness. MiR-203 overexpression in psoriasis suggests that miR-203 may be involved in an anti-inflammatory response because it targets both SOCS gene family members and pro-inflammatory cytokines.

## Key Points

- MiR-203 participates in the pathogenesis of psoriasis not only locally in skin lesions but also circulation, indicating that it might be involved in the systemic symptoms of the condition.
- MiR-203 overexpression in psoriasis may be a result of an anti-inflammatory response because it appears to affect cytokine signaling by targeting both SOCS gene family members and pro-inflammatory cytokines.
- Circulating miRNAs signature in plasma may improve psoriasis diagnosis and treatment outcomes.
- MiRNA-based therapies for psoriasis, as miRNAs are important players that influence various cellular processes by controlling a number of mRNAs and, consequently, their proteins.

## Introduction

Psoriasis is a chronic inflammatory dermatological illness affecting the skin, joints, or both. Its prevalence ranges from 2 to 3 percent worldwide and significantly lowers the quality of life (1). There are five different kinds of this condition, with psoriasis vulgaris accounting for about 90% of cases. Other types include guttate or eruptive psoriasis, inverted psoriasis, pustular psoriasis, and erythrodermic psoriasis (2).

Keratinocytes exhibit considerable proliferation and aberrant differentiation in psoriasis, while lymphocytes and neutrophils invade the epidermis. Tumor necrosis factor (TNF), interferon (IFN), transforming growth factor (TGF), interleukin (IL)-1, IL-17, and IL-22 are the primary pro-inflammatory cytokines involved in its development (3).

Additionally, it was shown that environmental variables and genetic vulnerability play important roles in the etiology of the illness. Recently, it has been proposed that the condition's etiology may entail genetic and epigenetic abnormalities, including genetic control by improperly expressed microRNAs (miRNAs) (4). MiRNAs have been discovered to play a key role in post-transcriptional gene silencing regulation, which may contribute to the etiology of the illness. Additionally, they have been demonstrated to operate as important mediators in a variety of cellular and developmental processes connected to various autoimmune illnesses, including but not limited to psoriasis (5, 6).

By attaching to the 3' untranslated region (UTR) of their target mRNAs, miRNAs, which are short, single-stranded, non-coding RNA molecules with 22 to 25 nucleotides, can adversely control gene expression. This causes the target mRNAs to degrade (7). The connection between psoriasis and miRNAs was first suggested in 2007 (8). In psoriasis patients, several miRNAs were identified to be inappropriately expressed in their peripheral blood or psoriatic lesions. When psoriasis samples were compared to normal skin biopsies, several miRNAs were investigated; nevertheless, the results were unclear (9–12).

Specifically, psoriasis displayed abnormal expression of miR-203 (8, 10, 13). It is believed to be a skin-specific signal that regulates cellular proliferation in a protein kinase C-dependent pattern because it is primarily generated by keratinocytes (8, 9). It was proposed that increased miR-203 expression in psoriasis lesions is correlated with diminished cytokine-signaling 3 (SOCS3) suppression and a resulting rise in the transcription factor (STAT-3), which is closely linked to the development of psoriasis (8). However, it was discovered that miR-203 might directly target and suppress the mRNAs of several cytokines, including TNF-α, IL-8, and IL-24, to lessen the inflammatory response (14, 15).

Furthermore, it was shown that miR-203 was associated with the reduction of tumor protein 63 (TP63), a crucial modulator of the basal cell “stemness,” which supported the hypothesis that miR-203 affected the proliferation of keratinocytes (16).

Finding aberrant expression of specific miRNAs in peripheral blood samples may improve the accuracy of some diagnoses and the efficacy of some treatments. The potential impact of psoriasis on a patient's quality of life highlights the importance of determining the precise immuno-molecular molecules implicated in its etiology. We conducted this study to evaluate miR-203 expression level in the plasma of psoriasis patients to investigate its role and target genes SOCS3, SOCS6, P63, TNF-α, IL-8, and IL-24 in the systemic pathogenesis of this disease and correlate these data with the disease course. To our knowledge, less is known about the specific role of circulating miR-203 in psoriasis.

## Subjects and methods

### Study participants

The 240 participants in the current study were divided into two groups: the cases group, which included 120 adult patients of both sexes with psoriasis, and the controls group, which included 120 matched, unrelated healthy volunteers. The personal and clinical characteristics were extracted from the patient's file, including the patient's age, sex, family history, body mass index (BMI), disease duration, age of commencement of disease, severity, and treatment. Dermatological examinations were performed on all patients to identify each lesion's size, location, pattern, and distribution. We used the Psoriasis Area and Severity Index (PASI) score to determine the severity of the condition.

### Selection of miR-203 target genes using bioinformatics tools

The miR-203 target genes were chosen utilizing a variety of miRNA-related platforms, including (a) microRNA.org (17) (b) miRDB, to find potential predicted and experimentally validated targets for miR-203 (18) Versions 2.0 of miRNAMap (19), 3.0 of miRGator (20) The human microRNA disease database (HMDD) version 2.0 (21), (e) DIANA-TarBase (22), (f) and (g) miRGate (23). At least two separate algorithms predicted each chosen target.

### Plasma samples and total RNA extraction

Three ml of fresh venous blood were drawn into vacutainer tubes containing the anticoagulant ethylene diamine tetraacetic acid (EDTA), then centrifuged to separate the plasma. Each patient's 100 l of plasma was mixed with 500 l of Qiazole reagent and kept at −80°C until further examination.

The manufacturer-recommended procedure was utilized to extract total RNA, including miRNAs, using the Qiagen miRNeasy mini kit (Qiagen, Catalog No. 217004). During the extraction procedure, an Eppendorf 5417C cooling microcentrifuge was employed. Purity and concentration of RNA were evaluated using NanoDrop 2000 1C at 260 and 280 nm absorbance (NanoDrop Tech., Inc. Wilmington, DE, USA). It was decided that values between 1.8 and 2.2 were sufficient for finishing the genetic study.

### Reverse transcription

The total RNA collected was transformed into cDNA by reverse transcriptase in the presence of oligo-dT priming using the MiScript II RT Kit (Qiagen, Catalog No. 218161). The RT reaction took place in a Veriti^TM^ 96-Well Thermal Cycler (Applied Biosystems, USA) at 37 °C for 1 h before being briefly incubated at 95 °C to inactivate the reaction.

### MiR-203 and its target genes expression analysis

The expression of miR-203 and its target genes were profiled using real-time PCR based on SYBR Green. For relative quantitative PCR, a template made from the cDNA premix was employed. Table 1 contains the primers for miR-203 and target genes (SOCS3, SOCS6, TP63, TNF-α, IL-8, and IL-24). To gauge the expression levels, a MiScript SYBR Green PCR Kit (Qiagen, Cat. No. 218076) was utilized. To facilitate data analysis using the CT approach, two endogenous controls—GAPDH for genes and SNORD68 for miR-203—were used. The “minimal information for publication of quantitative real-time PCR experiments (MIQE) guidelines” were followed during the qPCR experiment. Each reaction was run three times, with controls for each run of “No-RT” and “No-template.” Forty cycles of denaturation at 95 °C for 15 sec, annealing at 55 °C for 1 min, and elongation at 72 °C were performed during the run, which began at 95 °C for 5 min (1 min). The Livak method calculated fold changes based on the threshold cycle (CT) value using the relative expression = 2 ^−ΔΔCq^ equation (24).

**Table 1:** A list of the qRT-PCR primers.

**MiRNA/Gene**	**Forward primer**	**Reverse primer**
MiR-203	TAGTGGTCCTAAACATTTCAC	Universal Reverse Primers
SNORD68	GCCCCTGCGCAAGGATGAC	Universal Reverse Primers
SOCS3	AAGGCTCCTTTGTGGACTTCA	CGAATCGAAGTCTCCGTCCT
SOCS6	TGGTTTTCGCCTCGGAGTG	CCTTTGGAGCCATTTTCCGC
TP63	GAGACTGGACAGCCTTCACAA	GCTGGAAAACCTCTGGACTGA
TNF-α	CTGGGGCCTACAGCTTTGAT	GGCCTAAGGTCCACTTGTGT
IL-8	GGGTTGTGGAGAAGTTTTTGAAGA	TTCTGGTCATGAGTACAACAAACTC
IL-24	GGAGCTGCTTTCGCCAATTT	TCCTGAGCTTGCTGGCTAAA
GAPDH	GTCTCCTCTGACTTCAACAGCG	ACCACCCTGTTGCTGTAGCCAA

### Analysis of the miR-203 and its target genes under investigation for diseases, functions, and pathway enrichment

The disease, function, and pathway enrichment analyses of miR-203 and its target genes were performed using the Gene Analytics program (https://ga.genecards.org/) (25). The following criteria served as the foundation for the disease analysis score: (a) The total number of biomarkers directly linked to psoriasis divided by the number of understudied biomarkers implicated in the disease. (b) The nature and extent of the disease-biomarker relationship. The total scores of each matched biomarker are used to calculate the disease score. The findings were organized according to the order of the matching scores for pathways and GO keywords that matched the studied queries. Each biomarker's displayed score is a transformation (-log2) of the resulting *p*-value, with larger scores representing stronger matches.

### Statistical research

It was done using the Social Sciences (SPSS) Statistical Package for version 26.0 (IBM Corp., Armonk, NY). Using G^*^Power 3.1.9.2, the sample size and study power were estimated. For categorical data, frequencies and percentages were estimated; for continuous variables, means and standard deviations were calculated. The data profile was checked for normalcy and outliers. Correlation analysis was performed using Spearman's rank test, and a two-tailed *p*-value of 0.05 was deemed statistically significant. Biomarkers' diagnostic and prognostic utility was assessed using the area under the curve (AUC) *via* receiver operating characteristic (ROC) analysis.

## Results

### Baseline and clinical features for the study participants

The baseline and clinical characteristics of the study participants are shown in Table 2. The age range of the patients and the controls was 18 to 60. There was no statistically significant difference in age between the control group's age average of 38.4 ± 11.1 and the sick group's average age of 40.6 ± 12.9. There was no discernible difference between the patients, who comprised 65 percent non-smokers and 35 percent smokers, and the controls, who included 36.7 percent smokers and 63.3 percent non-smokers. Twenty percent of our psoriasis patients were obese, compared to eighty percent who were not. The mean BMI for patients was 26.55 ± 4.65 kg/m2, whereas the mean BMI for healthy controls was 27.78 ± 4.03 kg/m2. There was no statistical difference between the two groups. The average age when the sickness started was 34.35 ± 14.05, and the average length of time was 6.40 ± 6.55 years. The patients were divided into three subgroups based on the age at which the disease first manifested itself: (a) those with very early onset of psoriasis (vEOP) (up to 20 years), (b) those with middle early onset of psoriasis (mEOP) (between 21 and 40 years), and (c) those with late-onset of psoriasis (LOP) (over 40 years), which can be lifelong (46 patients). In total 42.5% of patients exhibited mild severity, 35% exhibited moderate severity, and 22.5 exhibited severe severity. According to the PASI score, the distribution of psoriasis patients ranged from 2.0 to 40.5, with a mean of 13.43 ± 8.66.

**Table 2:** Baseline and clinical characteristics of the research subjects.

**Variable**	**Cases** **(number = 120)**	**Controls** **(number = 120)**	***p*-value**
Age, mean (year)	40.6 ± 12.9	38.4 ± 11.1	0.163
Gender			
- Females	61 (50.8%)	54 (45%)	0.438
- Males	59 (49.2%)	66 (55%)	
Smoking			
- Non-Smoker	78 (65.0 %)	76 (63.3%)	0.79
- Smoker	42 (35.0%)	44 (36.7%)	
BMI	26.55 ± 4.65	27.78 ± 4.03	0.112
Obesity			
- Non-obese	80 (66.7%)	90 (75%)	0.201
- Obese	40 (33.3%)	30 (25%)	
Age of onset	34.35 ± 14.05		
- vEOP	30 (25.0%)		
- mEOP	44 (36.7%)		
- LOP	46 (38.3%)		
Severity of disease			
- Mild	51(42.5%)		
- Moderate	42 (35%)		
- Severe	27 (22.5%)		
Duration of disease	6.40 ± 6.55		
Family history			
- Positive	22 (18.3%)		
- Negative	98 (81.7)		
Treatment			
- No treatment	42 (35.0%)		
- On treatment	78 (65.0%)		
PASI	13.43 ± 8.66		

Data are presented as mean ± SD or number (percentage). *p*-values below 0.05 were deemed statistically significant. BMI, Body Mass Index; vEOP, Very early-onset psoriasis; mEOP, Middle-early onset psoriasis; LOP, Late-onset psoriasis; PASI, Psoriasis Area Severity Index.

### Psoriatic patients' expression patterns of miR-203 and its target genes

**Figure 1:**
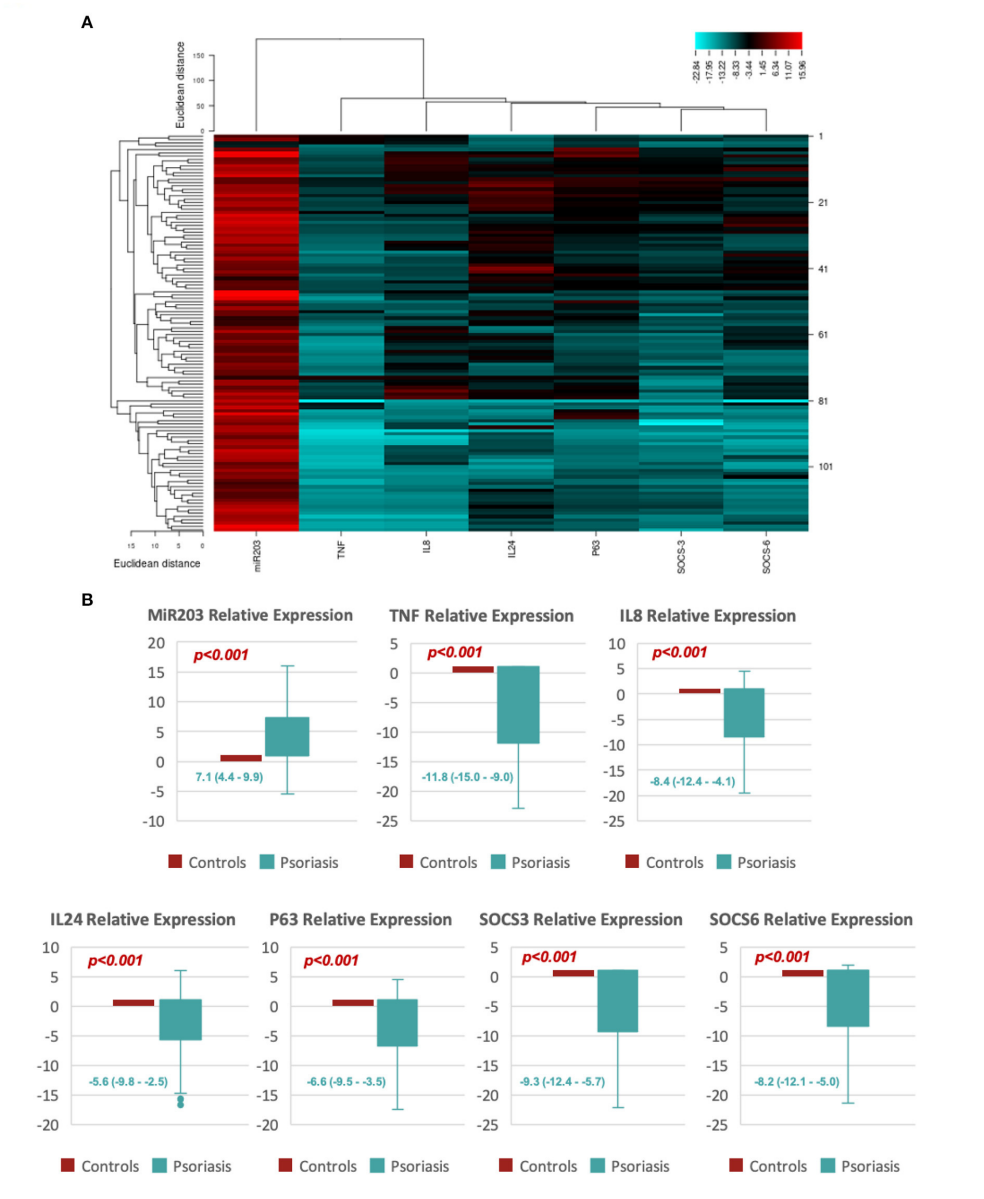
Shows the relative expression of MiR-203 and its target genes. **(A)** An example of a heat map showing the log fold change for each of the study's biomarkers (miR-203, SOCS3, SOCS6, TP63, TNF-α, IL-8, and IL-24). **(B)** Box plots showing the relative levels of miR-203 and its target gene expression. Values are shown as the median (Q1-Q3). The Mann-Whitney *U* test was utilized to determine the *p*-value.

Figure 1 shows that miR-203 expression levels in psoriatic patients were significantly higher than in healthy controls; 7.1 (4.4–9.9), *p* < 0.001. TNF-α [−11.8 (−15.0 to −9.0)], SOCS3 [−9.3 (−12.4–5.7)], SOCS6 [−8.2 (−12.1–5.0)], TP63 [−6.6 (−9.5–3.5)], IL-8 [−8.4 (−12.4–4.1)], and IL-24 [−5.6 (−9.8–−2.5)] all revealed significantly lower expression levels in psoriatic patients compared to healthy controls.

### MiR-203 and its target genes predictive significance by ROC curve analysis

**Figure 2:**
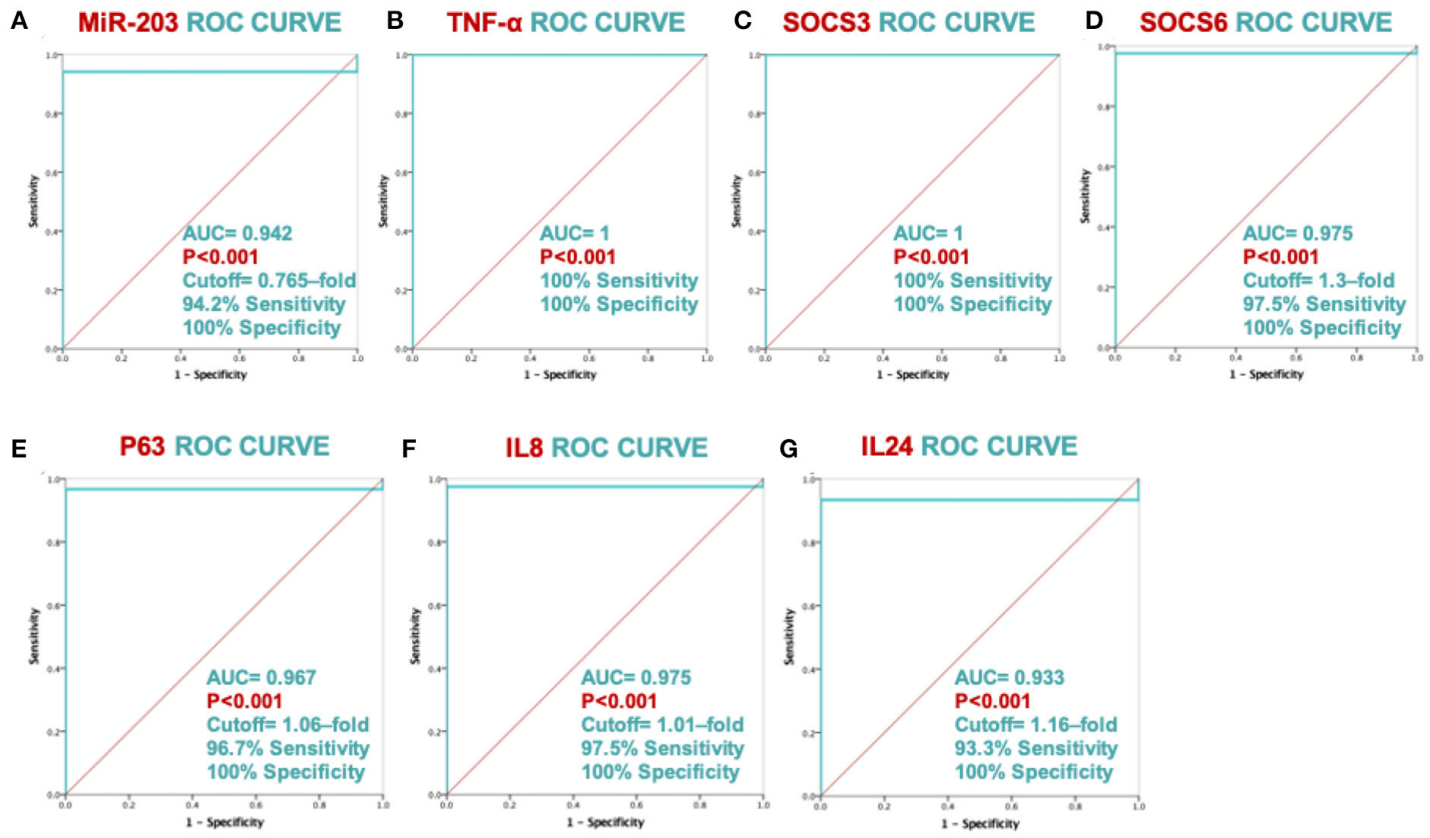
ROC curve analysis's ability to discriminate between MiR-203 and its target genes. The ROC curve with AUC and SE is displayed for each biomarker in graphs **(A–G)**.

ROC analysis showed significant discriminating power between psoriatic patients and healthy controls for all the study biomarkers, as shown in Figure 2; Plasma miR-203 levels of discrimination power scored 100% specificity and 94.2% sensitivity. Plasma TNF-α and SOCS3 discrimination power scored 100% sensitivity and 100% specificity. Plasma SOCS6, TP63, IL-8 and IL-24 levels discrimination power scored 97.5% sensitivity and 100% specificity, 96.7% sensitivity and 100% specificity, 97.5% sensitivity and 100% specificity, and 93.3% sensitivity and 100% specificity respectively.

### Correlation analysis of miR-203 and its target genes and the clinical features of the psoriatic patient

**Figure 3:**
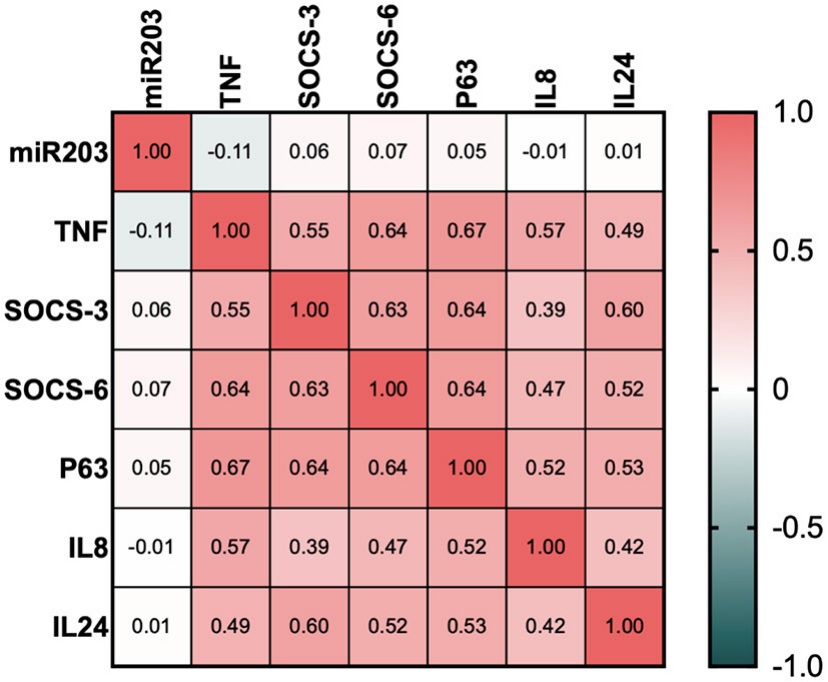
Correlation matrix for miR-203 and its target genes.

In Figure 3, a significant inverse correlation is displayed. All of miR-203's target genes were shown to have a strong negative connection. Even though all of the investigated genes SOCS3, SOCS6, TP63, TNF-α, IL-8, and IL-24 exhibited strong positive relationships.

Table 3 demonstrates the relationship between the patients' baseline and clinical data and the circulating biomarkers. A direct and significant connection between BMI and all target genes was observed. With *p* values of 0.05 and 0.019, the age-inverse significant connection for the TNF-α and IL-8 genes was seen. Family history and the SOCS3 gene showed a clear, significant link (*p* = 0.012).

**Table 3:** Correlation between the baseline and clinical information for research participants with psoriasis, miR-203, and its target genes.

		**Age**	**BMI**	**FH**	**Onset**	**Duration**	**Treatment**	**PASI**	**Severity**
MiR203	r (P_Corr_)	0.056	−0.105	−0.026	−0.081	−0.056	−0.024	0.042	0.062
	P_Ass_ value	0.390	0.106	0.775	0.382	0.541	0.797	0.647	0.504
TNF-α	r (P_Corr_)	−0.157*	0.127*	0.149	−0.082	−0.032	0.098	−0.101	−0.061
	P_Ass_ value	0.015	0.050	0.104	0.373	0.729	0.289	0.274	0.509
SOCS3	r (P_Corr_)	−0.118	0.132*	0.230*	−0.016	0.057	0.104	−0.103	0.010
	P_Ass_ value	0.068	0.042	0.012	0.860	0.533	0.258	0.263	0.910
SOCS6	r (P_Corr_)	−0.101	0.151*	0.108	−0.006	0.087	0.054	−0.043	−0.031
	P_Ass_ value	0.118	0.019	0.241	0.948	0.344	0.558	0.645	0.738
TP63	r (P_Corr_)	−0.105	0.166**	0.090	0.024	−0.033	0.165	−0.025	0.031
	P_Ass_ value	0.106	0.010	0.330	0.794	0.718	0.072	0.790	0.737
IL-8	r (P_Corr_)	−0.151*	0.163*	0.054	−0.105	0.026	0.026	0.000	0.013
	P_Ass_ value	0.019	0.011	0.557	0.252	0.779	0.781	0.999	0.888
IL-24	r (P_Corr_)	−0.124	0.140*	0.082	−0.030	−0.030	0.052	−0.070	−0.030
	P_Ass_ value	0.054	0.030	0.375	0.747	0.746	0.575	0.448	0.743

^*^Correlation is significant at the 0.05 level (2-tailed). ^**^Correlation is significant at the 0.01 level (2-tailed). Spearman's correlation analysis is expressed as a correlation coefficient (r) and *p*-values (P_Corr_). BMI, Body Mass Index; FH, Family History; Onset; The age of disease onset; PASI, Psoriasis Area and Severity Index.

### Analysis of miR-203 and its target genes' abundance in pathways, diseases, and functions

**Figure 4:**
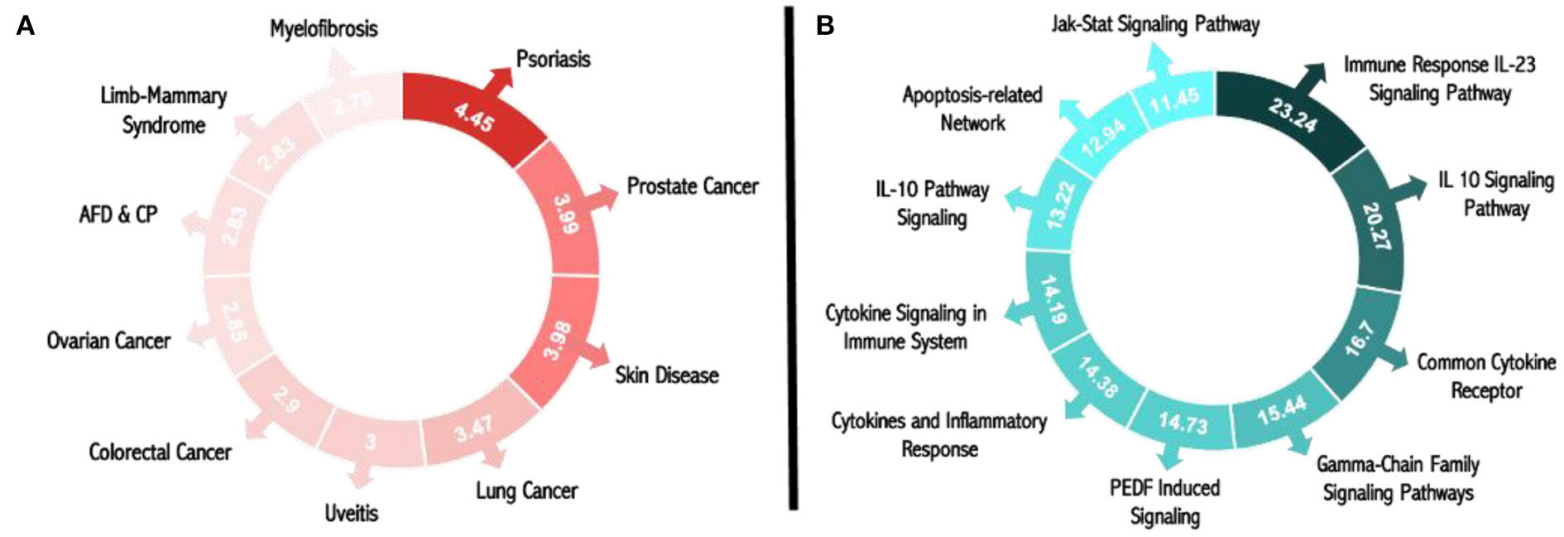
Matching scores for miR-203 and its target genes in pathways and illnesses. **(A)** Pathway matching score for psoriasis and all research biomarkers. **(B)** Score for all the study biomarkers that match diseases.

Figure 4A displays the results of the illness matching score; with a score of 4.45, psoriasis was determined to be the most common condition brought on by circulating miR-203 and its target genes. Additionally, the score demonstrates that miR-203 and its target genes are connected to numerous skin illnesses, as evidenced by the third-place ranking of skin diseases for this disease score with a value of 3.98. The genetic relationships of DE genes that were significantly up-or down-expressed in psoriasis patients relative to controls were included in these results, based on MalaCards sources.

The top 10 pathways associated with miR-203 and its target genes, according to the pathway matching score displayed in Figure 4B, are the immune response IL-23 signaling pathway, the IL-10 signaling pathway, the common cytokine receptor gamma-chain family signaling pathways, PEDF-induced signaling, cytokines, and inflammatory response, cytokine signaling in the immune system, interleukin-10 signaling, the apoptosis-related network associated with altered.

**Figure 5:**
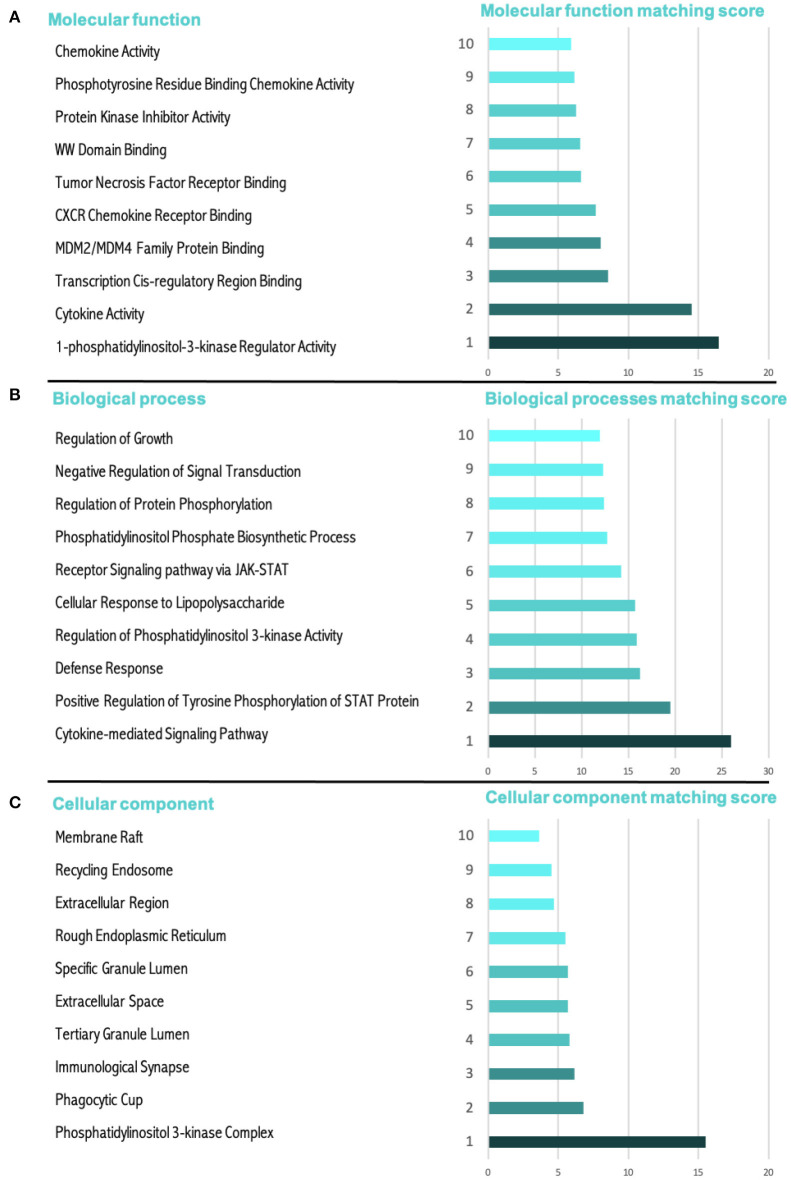
The gene ontology (GO) score for miR-203's target genes and miR-203 itself. **(A)** A cellular component and the score that matches it. **(B)** A molecular function and the score that matches it. **(C)** A biological process and the score that matches it.

Figure 5 shows the gene ontology analysis of all the study biomarkers. Based on the molecular function matching score, the top ten scores were found to be in the 1-phosphatidylinositol-3-kinase regulator activity, cytokine activity, transcription cis-regulatory region binding, MDM2/MDM4 family protein binding, CXCR chemokine receptor binding, tumor necrosis factor receptor binding, WW domain binding, protein kinase. The following were the top 10 biological processes for miR-203 and its target genes, as shown in Figure 5A. Figure 5B shows the signaling pathways that are cytokine-mediated, defense response, phosphatidylinositol 3-kinase activity regulation, receptor signaling pathway *via* JAK-STAT, cellular response to lipopolysaccharide, phosphatidylinositol phosphate biosynthesis, regulation of protein phosphorylation, negative regulation of signal transduction, and growth regulation. Last but not least, Figure 5C displays the ranking of the top 10 cellular components for miR-203 and its target genes as follows: phosphatidylinositol 3-kinase complex, phagocytic cup, immunological synapse, tertiary granule lumen, extracellular space, specific granule lumen, rough endoplasmic reticulum, extracellular region, recycling endosome, and membrane raft.

## Discussion

In 2007, researchers discovered a relationship between miRNAs and psoriasis (9). More than 250 miRNAs are differentially expressed in psoriasis lesions, most of which are found in peripheral blood or psoriatic skin (26). The miRNA profiles of unrelated psoriatic skin were compared to those of normal and healthy skin in certain studies (10, 12). Many miRNAs, including miR-203, have been shown to have a role in the immunopathogenesis of psoriasis (9, 11, 13). In order to gain a better knowledge of miRNAs role in the disease, the current study looked into the influence of miR-203 expression and its target genes SOCS3, SOCS6, TP63, TNF-α, IL-8, and IL-24 on the pathogenesis and clinical course of psoriasis.

As a control group, 120 psoriatic patients were compared to 120 age and sex-matched healthy participants. The mean age of the cases in the psoriasis group was 40.6 ± 12.9 years. In comparison, the control group was 38.4 ± 11.1 years in the current study, with no statistically significant difference between the two groups (*p* = 0.163). The psoriasis group had 49.2 percent males and 50.8 females, respectively, whereas the control group had 55 percent males and 45 percent females, with no statistically significant difference between the two groups (*p* = 0.258). Anani et al. (27) observed that psoriasis was more common in females (70%) than men among Egyptian patients, which is consistent with our findings (27). Some Western counties, such as Denmark (53.2 percent) and Minnesota, have also observed a female predominance (28–30). Nepal, the Maghreb, and Malaysia, on the other hand, reported a male preponderance (56%) (53.7 percent, 55.7 percent, and 56.6 percent, respectively) (31–33). On the other hand, some scholars attributed the variation to racial variations among the various populations surveyed.

Smokers accounted for 35 percent of the psoriasis cases. This result is in line with Ortiz et al. (34), who discovered a link between smoking and metabolic syndrome. Psoriasis is substantially linked to smoking (34). Nicotine binds to nicotinic acetylcholine receptors on DCs, macrophages, endothelial cells, and keratinocytes in the skin, enhancing the synthesis of pro-inflammatory cytokines like IL-12, IL-1, and TNF-α (35), which may lead to local skin synthesis of A-SAA (34). The metabolic syndrome is known to be linked to psoriasis (36).

In terms of BMI, there was no statistically significant difference between the two groups in terms of mean BMI. This finding is consistent with the findings of Genc et al. (37), who found no significant difference in BMI between psoriasis and control groups (*p* > 0.05) (37). Haberka and colleagues also demonstrated no differences in BMI between the psoriatic patients and the control group (38).

In the psoriasis group, the average age of onset of psoriasis was 34.35 ± 14.05 years, while the average disease duration in the psoriasis group was 6.40 ± 6.55 years. According to Ali et al. (39), the duration of psoriasis ranges from one to 35 years (mean of 10.59 years) (39). According to Genc et al. (37), psoriasis patients' disease duration ranged from one to 40 years (mean: 13.8 12.0 years) (37).

Regarding psoriasis family history, regarded as one of the risk factors for the disease's development (40), there were 22 cases in our study with a positive family history of the condition (18.3 percent). El-Komy et al. (41) observed similar findings, revealing that 10.5 percent of their patients had a positive family history (41). This result is lower than the percentages reported in studies conducted in Italy, Spain, the Maghreb, China, and Malaysia, which found 45.9%, 40.7 percent, 28.6 percent, 23.1 percent, and 23.1 percent of patients, respectively (32, 42–45).

This finding could be due to cultural and social issues, such as patients denying the disease runs in their family. Such a denial may provide patients with some reassurance in avoiding social stigmatization and exclusion, a fear exacerbated by the local culture of the “ideal partner,” to the point where psoriasis patients have formed social network organizations to socialize for marriage purposes (46, 47). More research into the rate of family history is required for more accurate data, ideally by family physicians.

MiR-203 is a skin-specific miRNA that is overexpressed solely in psoriatic keratinocytes and plays a role in psoriasis angiogenesis and keratinocyte differentiation (1). In the current study, psoriatic patients' plasma had substantially higher levels of miR-203 expression than controls; 7.1 (4.4–9.9), (*p* < 0.001). This result was supported by a study by Xiao et al. (48), who looked at the miR-203 expression in psoriasis lesions and non-lesion tissues and found that miR-203 expression was considerably higher in psoriasis lesions (48). In a similar vein, two prior investigations found that miR-203 was a 5.86- and 2.02-fold increase in psoriatic skin compared to normal skin, respectively (9, 13).

MiR-203 expression is dramatically elevated in mice and HaCaT cells treated with IL17, according to Xu and colleagues. In addition, VEGF levels in the ears of IL-17-stimulated mice and the supernatant of IL17-treated HaCaT cells were shown to rise. These findings suggest that IL-17 can cause VEGF expression to increase and that miR-203 may mediate this effect. Furthermore, inhibition of miR-203 reversed the IL-17-induced increase in VEGF secretion and inhibited the IL-17-induced activation of JAK2/STAT3 signaling, according to the findings (49). The top 10 pathways related to miR-203 and its target genes, according to our pathway enrichment analysis, are immune response IL-23 signaling pathway, IL-10 signaling pathway, common cytokine receptor gamma-chain family signaling pathways, PEDF induced signaling, cytokines, and inflammatory response, cytokine signaling in the immune system, interleukin-10 signaling, apoptosis-related network due to altered notch3 in ovarian cancer and Jak-Stat signaling pathway.

TNF-α [−11.8 (−15.0 to −9.0)], SOCS3 [−9.3 (−12.4–5.7)], SOCS6 [−8.2 (−12.1–5.0)], TP63 [−6.6 (−9.5–3.5)], IL-8 [−8.4 (−12.4–4.1)], and IL-24 [−5.6 (−9.8–2.5)] all revealed significantly lower expression levels in psoriatic patients compared to controls. Gupta et al. (50), on the other hand, found that in psoriasis patients, the genes coding for IFN (interferon), c-myc, IL-6, and IL-8 were greatly enhanced (50). The difference between the two studies could be explained by the type of material used, as they measured expression in the skin, whereas we measured it in plasma. Furthermore, according to Wei et al. (51), variations in expression level can occur due to differences in the skin layer studied (51).

SOCS proteins are activated by growth factors or cytokines and regulate the length and amplitude of inflammatory responses by inhibiting JAK proteins in a negative feedback loop (52). SOCS1 and SOCS3 proteins are important regulators of the JAK2/STAT3 signaling pathway in previous research (53–55). Our findings corroborated those of Xu et al. who found that expression was considerably reduced when HaCaT cells were stimulated with IL-17, SOCS1, and SOCS3 (49).

TP63 is required to form epidermis and other stratified epithelia, and it functions as a molecular switch between proliferating basal cells and suprabasal cells (56–59). MiR-203 has been proven to target TP63 directly (16, 60). TP63 expression was downregulated in the suprabasal layers of the epidermis, according to Wei et al. (51), although TP63 and SOCS3 were preferentially expressed in the basal layer (51).

MiR-203 had a statistically significant strong inverse connection with SOCS3 (*p* < 0.001, *r* = −0.624) and SOCS6 (*p* < 0.001, *r* = −0.583) in the current investigation. This finding was supported by Sonkoly et al. (61), who found that the upregulation of miR-203 in psoriasis lesions is associated with the downregulation of SOCS3 (61).

In the same scenario, Xu et al. found an inverse relationship between miR-203 and SOCS3 expression in HaCaT cells activated by IL-17. These findings imply that SOCS3 is a direct target of miR-203 and may play a role in the IL-17-induced VEGF production process in HaCaT cells (49). The suppression of SOCS3 by miR-203 adds to the complexity of SOCS3 regulation, which could have substantial ramifications for keratinocyte functions in both developing and adult skin (51).

The BMI of the patients and all target genes were directly and significantly correlated in this study. These results were consistent with those of an earlier study by Eikelis et al. who discovered that hepatic and visceral fat miR-132 expression was associated with BMI and that miR-132 reduction may be a target for the regulation of liver lipid homeostasis and control of obesity-related blood pressure (62).

The IL-31/IL-33 axis is a potential mechanism of inflammation in autoimmune and allergy illnesses by Murdaca et al. (63). For the emergence of allergic inflammation, such as in asthma, activating the IL-33/ST2-involving Th2/IL-31 immune response is particularly important. The dose of these cytokines may one day help diagnose, stage, and monitor the therapy's effectiveness in various allergy and autoimmune disorders. The development of new therapeutic strategies for treating these inflammatory disorders may depend heavily on the pharmacological control of IL-33/ST2 activity.

Finally, our findings demonstrate that miR-203 contributes to the pathogenesis of psoriasis not just locally in skin lesions but also in circulation, suggesting that it can play a role in the disease's systemic symptoms. MiR-203 appears to influence cytokine signaling by targeting both SOCS gene family members and pro-inflammatory cytokines, implying that miR-203 overexpression in psoriasis may be part of an anti-inflammatory response. Our results suggest that miRNA-based therapeutics in psoriasis can be a more tailored and effective option than existing traditional medications with mono protein targeting action since miRNAs are major players that affect various cellular processes through the regulation of several mRNAs and, thereby, their proteins.

## Conclusion

Our findings demonstrate that miR-203 contributes to psoriasis pathogenesis not just locally in skin lesions but also in circulation, suggesting that it may possibly be involved in the illness' systemic symptoms. Because miR-203 targets both members of the SOCS gene family and pro-inflammatory cytokines, it is possible that miR-203 is implicated in the anti-inflammatory response in psoriasis.

## Data availability statement

The original contributions presented in the study are included in the article/supplementary material, further inquiries can be directed to the corresponding author/s.

## Ethics statement

The studies involving human participants were reviewed and approved by the Suez Canal University, Faculty of Medicine, Ethics Committee in Ismailia, Egypt (Approval No. 4502) and conducted according to the Declaration of Helsinki's guidelines. Informed consent was obtained from all individual participants included in the study. The patients/participants provided their written informed consent to participate in this study.

## Author contributions

SM, HA, NT, and GB designed the study. NT collected the clinical data. MM collected the patients' samples. HA, MM, and SM carried out the experiments. SM, HA, WN, SSA, MW, SMA, and MM analyzed and interpreted the patient data. All authors discussed the results, contributed to the final manuscript writing, and approved it.

## Conflict of interest

The authors declare that the research was conducted in the absence of any commercial or financial relationships that could be construed as a potential conflict of interest.

## Publisher's note

All claims expressed in this article are solely those of the authors and do not necessarily represent those of their affiliated organizations, or those of the publisher, the editors and the reviewers. Any product that may be evaluated in this article, or claim that may be made by its manufacturer, is not guaranteed or endorsed by the publisher.
